# Magnetite Synthesis in the Presence of Cyanide or Thiocyanate under Prebiotic Chemistry Conditions

**DOI:** 10.3390/life10040034

**Published:** 2020-04-02

**Authors:** Rafael Block Samulewski, Josué Martins Gonçalves, Alexandre Urbano, Antônio Carlos Saraiva da Costa, Flávio F. Ivashita, Andrea Paesano, Dimas Augusto Morozin Zaia

**Affiliations:** 1 Departamento de Química, Universidade Estadual de Londrina, CEP 86057-970 Londrina, PR, Brazil; blockeness@gmail.com; 2 Departamento de Química Fundamental, Universidade de São Paulo-USP, CEP 05508-000 São Paulo, SP, Brazil; josuemartins@usp.br; 3 Departamento de Física-CCE, Universidade Estadual de Londrina, CEP 86057-970 Londrina, PR, Brazil; aurbano@uel.br; 4 Departamento de Agronomia-CCA, Universidade Estadual de Maringá, 87020-900 Maringá, PR, Brazil; antoniocscosta@gmail.com; 5 Departamento de Física-CCE, Universidade Estadual de Maringá, 87020-900 Maringá, PR, Brazil; fivashita@gmail.com (F.F.I.); paesano@wnet.com.br (A.P.J.)

**Keywords:** cyanide, magnetite, prebiotic chemistry, thiocyanate

## Abstract

Magnetite is an iron oxide mineral component of primitive Earth. It is naturally synthesized in different ways, such as magma cooling as well as olivine decomposition under hydrothermal conditions. It is probable magnetite played a significant role in biogenesis. The seawater used in the current work contained high Mg^2+^, Ca^2+^ and SO_4_^2−^ concentrations, unlike the seawater of today that has high Na^+^ and Cl^−^ concentrations. It is likely that this seawater better resembled the ion composition of the seas of the Earth from 4 billion years ago. Cyanide and thiocyanate were common molecules in prebiotic Earth, and especially in primitive oceans, where they could act on the magnetite mechanism synthesis via Fe^2+^ interaction. In this research, magnetite samples that were synthesized under prebiotic conditions in the presence of cyanide or thiocyanate, (both with and without artificial seawater), showed that, besides magnetite, goethite and ferrihydrite can be produced through different Fe^2+^-ion interactions. Cyanide apparently acts as a protective agent for magnetite production; however, thiocyanate and seawater 4.0 Gy ions produced goethite and ferrihydrite at different ratios. These results validate that Fe^3+^ oxides/hydroxides were possibly present in primitive Earth, even under anoxic conditions or in the absence of UV radiation. In addition, the results show that the composition of water in early oceans should not be neglected in prebiotic chemistry experiments, since this composition directly influences mineral formation.

## 1. Introduction

The period close to 4 billion years ago was significant with respect to in the number of different types of minerals, which increased due to the evolution of igneous rocks and the weathering caused by the large amount of water at the time. It is believed that during that geological era, the number of mineral species increased from approximately 60 to 500 due to the transformation of primary chondrites, such as olivine, to a wide range of clays, zeolites, and transition metal oxides and hydroxides. The main metallic minerals that covered the crust of the primitive Earth were iron oxides and hydroxides, and as such it is assumed that these minerals played an important role in primitive biogenesis [1,2,3,4].

Magnetite is an iron oxide with mixed iron valency that was present in primitive Earth [3,4]. This prebiotic iron oxide may have been synthesized by magma cooling, olivine decomposition under hydrothermal conditions, or pH increases in Fe^2+^ solutions generated through weathering [5,6,7]. In the latter case, thermodynamically instable hydroxide (Fe(OH)_2_) is produced and, even in the total absence of oxygen or other oxidizing agents, Fe(OH)_2_ is transformed directly into magnetite. Under prebiotic conditions, any molecule that can accept electrons is part of the magnetite-forming process due to the precipitation of Fe(OH)_2_ [8]. This statement is extremely curious for prebiotic chemistry because it means that unlikely molecules such as water, nitrogen, and carbon oxides may have acted as oxidant agents that led to the formation of other building blocks such as ammonia, formaldehyde, and lipids [9,10,11,12,13,14]. Schrauzer and Guth showed that synthesis of magnetite in the presence of carbon monoxide as an electron acceptor leads to methane production [8]. In addition, other molecules must also have been important in the magnetite synthesis process under prebiotic conditions.

Bassez suggested that the presence of oxygen or ultraviolet radiation is not necessary for Fe^2+^ to Fe^3+^ oxidation. According to the author, compounds containing Fe^3+^ can be formed under anoxic conditions at temperatures ranging from 300 to 350 °C, pressures between 10 and 25 MPa, and pH ranges between 9.5 and 14 [15]. These conditions have been found in various hydrothermal sources and ferrihydrite, hematite, goethite, and lepidocrocite have been formed in these environments [16].

In another study, FischerTropsch synthesis (FTS) was conducted with magnetite as a catalyst to produce abiotic hydrocarbon under simulated prebiotic conditions [17]. From a geological perspective, a hydrothermal reaction between molecular hydrogen and CO or CO_2_ with mineral iron oxides as catalyst (e.g., magnetite) produces CH_4_ and other carbon-reduced molecules. Various authors have found a diversity of carbon chain products including alkanes, alcohols, and alkanoic acids when magnetite acts as a catalyst, simulating FTS under prebiotic hydrothermal conditions [17,18,19,20,21]. Rao et al. used Mössbauer spectroscopy during FTS of hydrocarbon production with magnetite as the catalyst under an H_2_/CO flow system, demonstrating that octahedral sites of mineral are linked to carbon species adsorption and that cation-deficient magnetite increases CO conversion [17]. On the other hand, Datye at al. have suggested that magnetite acts as a catalyst only when there is carbide stabilization under its surface and that carbide is responsible for the growth of carbon chains, under H_2_/CO flow systems [18]. 

Cyanide and thiocyanate are two important ions in prebiotic chemistry that have been detected in several prebiotic simulations as well as in comets, asteroids, and stardust. These ions are considered excellent building blocks and are directly linked to the synthesis of molecules such as amino acids, purines, and pyrimidines [22,23,24,25,26,27,28]. Since these two ions can produce complexes with Fe^2+^, their presence under magnetite synthesis conditions is important to understanding if they can interfere in mineral formation. In addition, the two molecules could act as electron receptors from Fe(OH)_2_ and lead to the formation of molecules with prebiotic relevance.

One factor commonly overlooked in prebiotic experiments is the composition of the solutions. The majority of prebiotic chemistry experiments have been carried out in distilled water or in an NaCl solution [29]. It should be noted that distilled water or NaCl solutions do not resemble the composition of the seas of the Earth of 4 billion years ago. Thus, based on the work of Izawa et al. (2010), who performed leaching experiments on Tagish Lake meteorites (obtaining the following order of cations: Mg^2+^ > Ca^2+^ >> Na^+^ ≈ K^+^, and anions: SO_4_^2−^ >> Cl^−^ [30]), we suggested an artificial seawater containing high Mg^2+^, Ca^2+^, and SO_4_^2−^ concentrations—unlike the seawater today, which has high Na^+^ and Cl^−^ concentrations [29]. However, if the prebiotic sea had a high quantity of MgSO_4_, over time this would have evolved into a sea with a high quantity of MgCl_2_, which is very different from the high quantity of NaCl in the present-day composition [30]. To get around this problem Izawa et al. suggested that the evolution of seawater occurred in two steps: (1) before 3.7 Ga, dissolution of minerals was a major process, and (2) after 3.7 Ga, the weathering process of the crust was the major process, reaching the composition of modern seawater by ~3.3–3.0 Ga. In addition, there would have been several Mg^2+^ sequestering processes, such as the formation of clay minerals, dolomites, among others [31]. It should be noted that the work of Izawa assumes that exogenous minerals (meteorites) released several ions after being leached by water. However, Boehnke and Harrison (2016) have raised some issues about whether the later heavy bombardment occurred [32]. In addition, Breuer (2018) suggested two hypotheses for the origins of water, the atmosphere, and the Earth’s crust; (1) outgassing from the interior, or (2) late delivery from comets or asteroids [33].

In the present work, the effects of cyanide and thiocyanate, as well as synthetic seawater 4.0 Gy, were studied on the synthesis of magnetite under prebiotic chemistry conditions. It should be noted that there have been no reports in the literature about the influence of these two ions on the synthesis of magnetite under prebiotic chemistry conditions. Thus, these experiments are interesting from a prebiotic point of view, and make it possible to elucidate the importance of these ions in the formation of minerals and/or organic molecules.

## 2. Materials and Methods

### 2.1. Materials

All reagents utilized were chemical grade and were used without previous purification. Each synthesis was performed at least six times.

### 2.2. Methods

Magnetite samples were synthesized using the modifications of the methodology described by Schwertmann and Cornell [34]. All samples were synthesized using the same methodology; however, reagents and solutions differed according to the synthesis summary outlined in Table 1. Control experiments were also performed in order to show that contaminants or oxygen were not interfering in the compounds obtained (Tables S1 and S2). The experiments were not run in the dark. All solutions were previously prepared in nitrogen inert atmosphere systems. For standard samples of magnetite synthesis (MGP), 5.72 g (30 mmol) of ferrous chloride heptahydrate (FeCl_2_·7H_2_O) was dissolved in 60 mL of previously deaerated ultrapure water and the resulting solution was heated to 90 °C (SOLUTION 1). A second solution was prepared using 4.49 g (80 mmol) of potassium hydroxide (KOH) and 0.646 g (6.5 mmol) of potassium nitrate (KNO_3_) dissolved in 25 mL of deaerated ultrapure water and heated to 90 °C (SOLUTION 2). After both solutions reached 90 °C, SOLUTION 2 was added slowly (approximately 4 mL per minute) to SOLUTION 1 under constant stirring until a dark precipitate formed. After complete addition, the resulting dispersion was stirred for an additional 40 min under an inert atmosphere, then placed under refrigeration at 5 °C for 24 h. After this time, the black solid was filtered and washed three times with ultrapure water, frozen, and finally lyophilized to obtain the dry solid. Modifications in the methodology were carried out through changes in SOLUTION 1, since the presence of potassium hydroxide in SOLUTION 2 would cause calcium and magnesium precipitation. Changes in the solution composition were basically due to the insertion of cyanide or thiocyanate ions and the replacement of ultrapure water with artificial seawater 4.0 Gy as described by Zaia (Table S3) [30]. 

Infrared spectra were obtained using a Bruker spectrophotometer model Vertex 70 with Attenuated Total Reflectance (ATR) accessory, using 32 scans with a 2 cm^−1^ resolution and a working window between 4000 and 400 cm^−1^.

X-ray diffractograms were obtained using Shimadzu^®^ XRD-6000 equipment, with Co-Ka radiation (λ = 1.78901 Å) and an iron filter operating at 30 mA and 40 kV. The scanning parameters were set at 0.02 2θ with a time of 0.6 s and a scan window of 5.0 to 70.0 2θ. The diffractograms were analyzed using the FullProf Suite through Rietveld’s refinement method.

Transmission electronic microscopy (TEM) images were obtained on JEOL JEM-2100 equipment with an acceleration voltage of 200 kV. The samples were prepared by dispersing magnetite samples in ultrapure water then adding the solution to a copper grid and covering it with an ultra-fine carbon film (TedPella). The energy-dispersive spectroscopy (EDS) was performed by the Oxford^®^ X-MaxN 80T instrument coupled to the microscope. The diffraction fringes were analyzed using GIMP image software 2.10.8 through pixel counts in high resolution images.

Mössbauer spectroscopy measurements were performed in a spectrometer calibrated with a metallic iron absorber, operating in constant acceleration mode. Measurements were performed using ^57^Fe as a nuclear probe and the 14.4 KeV radiation emitted by a 57Co (Rh) source. All measurements with this nuclear probe were performed at room temperature. The spectra were adjusted by a numerical routine applying the Lorentzian model, using the least squares criterion to calculate the parameters.

The point of zero charge (pH_PZC_) was determined from suspensions of the samples according to the method described by Uehara [35]: 50 mg of the magnetite samples were added to two 2 mL Eppendorf tubes, and 125 μL of 1.0 mol L^−1^ KCl solution was added to one tube and 125 μL ultra-pure water to the other. Both tubes were shaken for 30 min and after 24 h the pH was measured. Everything was prepared in triplicate. The pH_pzc_ was calculated using the following equation: pH_pzc_ = 2 pH_KCl (1.0 mol/L)_ − pH_ultra pure water_.

Cyanide and thiocyanate quantification were performed in the UV-Visible region using Spectrum SP2000-UV equipment. Determination of cyanide was performed through modification of the methodology described by Scoggins, which uses the formation of nickel complex with a cyanide binder. Nickel reactive solution was prepared using a concentration of nickel chloride 1.0 10^−3^ mol L^−1^ and ammonium hydroxide 0.5 mol L^−1^ [36,37]. Standard cyanide solutions were prepared using potassium cyanide in concentrations from 0.25 to 600 mg L^−1^. For each 1 mL of standard solution, 5 mL of the nickel reactive solution was added and the resulting solutions were allowed to stand for 10 min. After this time, the absorbance of the samples was measured at the 267-nm wavelength using a 1-cm quartz cuvette. Quantification of thiocyanate ions was performed using a modification of the method described by Martins et al., which is based on the formation of a complex between iron (III) and the thiocyanate ion [38]. The reactive iron solution was prepared using 2.410 g of FeCl_3_·6H_2_O dissolved in 10% HNO_3_ solution. Standard thiocyanate solutions were prepared using potassium thiocyanate dissolved in ultrapure water at concentrations from 3.0 to 40 mg L^−1^. For each 0.5 mL of standard solution, 1.0 mL of the iron reactive solution was added, diluted to 10 mL with ultrapure water and allowed to stand for 10 min. After this time, the absorbance of the samples at the 460-nm wavelength was measured using a 1 cm quartz.

For cyanide and thiocyanate adsorption assays, 50 mg of synthesized magnetite was suspended in 2.0 mL of 720 mg L^−1^ cyanide or thiocyanate solutions prepared in ultrapure water, seawater 4.0 Gy, KCl 0.1 mol L^−1^, and KCl 1.0 mol L^−1^. The samples received constant stirring at room temperature for 1, 24, and 48 h in an inert atmosphere. All samples were performed in triplicate. After the end of each period, the samples were centrifuged at 6000 rpm and the cyanide and thiocyanate quantification were performed as previously described.

## 3. Results

**Figure 1:**
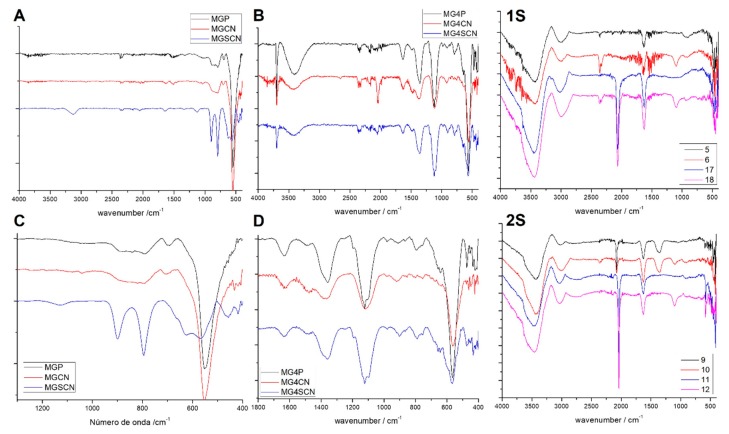
Fourier Transform Infrared-Attenuated Total Reflectance-FTIR-ATR spectra of all solid samples obtained: (**A**). Spectra of ultrapure water synthesis products; (**B**). Spectra of seawater 4.0 Gy synthesis products; (**C**). Magnification of 400 to 1300 cm^−1^ region from A; (**D**). Magnification of 400 to 1800 cm^−1^ region from B. (Sample codes according to Table 1). FTIR-ATR spectra of precipitates of control experiments: (**1S**). Spectra of Fe^2+^ solution in ultrapure water or in seawater 4.0 Gy plus KOH and Fe^2+^ solution in ultrapure water or in seawater 4.0 Gy plus KOH plus KSCN; (**2S**). Spectra of Fe^2+^ solution in ultrapure water or in seawater 4.0 Gy plus KCN plus KNO_3_ and Fe^2+^ solution in ultrapure water or in seawater 4.0 Gy plus KCN plus KOH (Sample codes according to Table S1).

Control synthesis were not run in the dark and they did not show the production of hydroxy/oxide iron compounds (Figure 1 and Table S2). Thus, the light does not influence the production of hydroxy/oxide iron compounds. Braterman and cols showed that the highest Fe^3+^ formation rate occurred with UV radiation at 217 nm [13,14]. Using the highest Fe^3+^ formation rate obtained by Braterman and cols and the reaction time of 30 min, we calculated that only 0.16% of all Fe^2+^ in solution would be oxidized. It should be noted that under condition in which all experiments were performed this value was probably much lower, because the wavenumber radiation that could reach the reaction vessel is much higher than the one used by Braterman and cols. In addition, the control experiments showed that for the synthesis of magnetite, KOH and KNO_3_ are necessary in solution. All syntheses produced dark solids with different black shades; left spectra for ultrapure water synthesis and right for seawater 4.0 Gy (Figure 1). MGP and MGCN samples showed bands at 557, 697, and 840 cm^−1^ due to the Fe–O and Fe–OH stretching of magnetite. MGSCN and MG4SCN showed two bands at 795 and 989 cm^−1^ typical for goethite formation. Samples synthesized in seawater 4.0 Gy solution showed many bands that did not belong to the magnetite phase. MG4P, MG4CN, and MG4SCN samples showed bands at 1100 and 3690 cm^−1^ characteristic of gypsum formation (CaSO_4_ 2H_2_O). In addition, two other bands at 1355 and 1476 cm^−1^ could be attributed to symmetric and asymmetric stretch of CO_3_^2−^ typical of ferrihydrite formation. These samples also showed a large band at 3400 cm^−1^ that could be attributed to O–H stretch from the mineral surface. The MG4CN sample showed a tiny band at 2042 cm^−1^ due to the cyanide ion.

**Figure 2:**
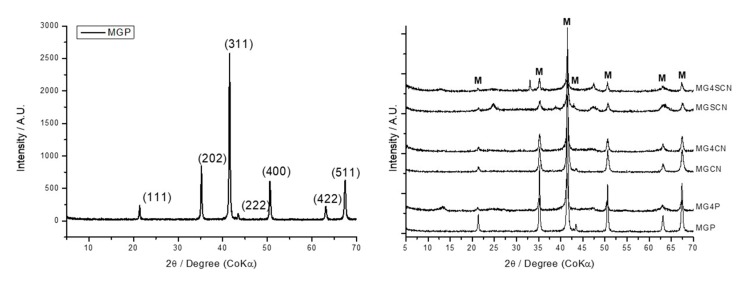
XRD pattern of MGP sample (**left**) and XRD patterns obtained for all solid samples (**right**).

Figure 2 presents the XRD pattern for the MGP sample (left) and sequential XRD patterns of all solid samples obtained (right). For all samples, magnetite diffraction peaks are visualized as described for the MGP sample. Peaks at 2θ = 21.30, 35.13, 41.44, 43.37, 50.50, 62.92, and 67.22 refer to diffraction plates 111, 202, 311, 222, 400, 422, and 511, respectively. MG4P, MG4CN, and MG4SCN samples presented a large peak at 12.9° 2θ respective to the gypsum presence. MG4P, MGSCN, and MG4SCN samples showed peaks at 47 and 25.2 due to goethite formation. A typical peak of sylvite, a KCl mineral, was observed in the MG4SCN sample by the emergence of a 33.2θ peak.

Rietveld parameters for XRD patterns are presented in Table 2. For all samples Chi square (χ^2^) values lower than 5 and a Rietveld parameter (R_wp_) around 20% indicates good correspondence with experimental data. MGP, MGCN, and MG4CN samples showed only magnetite. Goethite was present in MG4P, MGSCN, and MG4SCN. Gypsum appeared in the MG4P and MG4SCN samples. Sylvite was found only in MG4SCN (Figure 2 and Table 2).

The data obtained from Mössbauer spectra from all solid samples obtained at room temperature can be seen in Table 3. For calculation of parameters, the least squares method was used, allowing radiation absorption/emission of the Lorentzian set-up. As there was magnetite phase identification in all samples, parameters were adjusted using the MGP sample data as standard.

**Figure 3:**
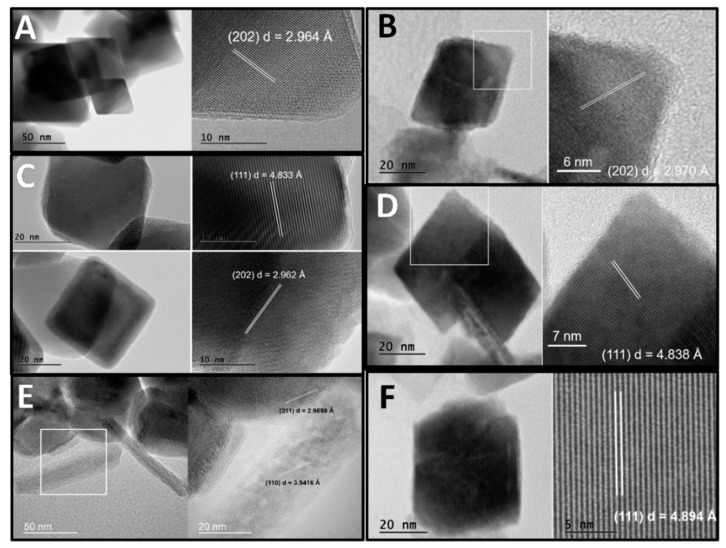
TEM images of all solid samples, with magnification of selected areas to show diffraction fringes and their respective interplanar distances (**A**—MGP; **B**—MG4P; **C**—MGCN; **D**—MG4CN; **E**—MGSCN; **F**—MG4SCN).

Selected transmission electron microscopy (TEM) images of all samples can be seen in Figure 3. Octahedral magnetite crystallites were present in all samples with different crystallinity. For all samples, image magnification was used for interplanar distance calculation of the diffraction fringe found. Interplanar distances of different diffraction planes can be seen on the right side of Figure 3.

Supernatants of MGCN, MG4CN, MGSCN, and MG4SCN samples did not demonstrate significant changes in cyanide or thiocyanate concentrations from the initial values, meaning that they were not adsorbed during the synthesis of the minerals. In addition, for all samples, adsorption of cyanide and thiocyanate onto magnetites was performed with an initial concentration of 720 mg L^−1^ at pH 7.0. Cyanide and thiocyanate solutions were prepared in ultrapure water, seawater 4.0 Gy, KCl 0.1 mol L^−1^ and KCl 1.0 mol L^−1^. The samples were kept under constant stirring at room temperature for 1, 24, and 48 h in an inert atmosphere. No significant adsorption of cyanide or thiocyanate was observed in any experiment. Although the experiments were also performed at pH values from 3.5 to 9.0, non-significant ion adsorption was observed.

## 4. Discussion

For the MGP and MGCN samples, the FT-IR spectra exhibited typical magnetite bands, indicating that cyanide did not have any influence on mineral formation (Figure 1) [34]. In contrast, for the MGSCN and MG4SCN samples, as described by other authors, thiocyanate induced goethite formation via Fe^II^-S interaction (Figure 1) [27,39,40,41,42,43,44,45]. This is evidence that the composition of primitive oceans had a lot of influence on the formation of iron minerals under prebiotic conditions. The FT-IR spectra of the samples synthesized using seawater 4.0 Gy showed many more bands when compared to the FT-IR spectra of the MGP samples (Figure 1). It seems that seawater 4.0 Gy ions have an effect on the formation of different mineral phases. For all these samples, the ferrihydrite phases found at the 1355 and 1476 cm^−1^ bands were due to the carbonate symmetric stretch that is typical of ferrihydrite mineral formation. Gypsum was also observed through the appearance of a thin band at 3690 cm^−1^ [41,46]. For the MGSCN and MG4SCN samples, goethite bands were observed at 795 and 898 cm^−1^, but the same bands were not found in the MG4CN or MGCN samples. Somehow, the presence of cyanide did not lead to goethite formation, preventing goethite mineral phase formation. It should be noted that the results obtained from the FT-IR spectra were confirmed by XRD patterns (Figure 2 and Table 2) and Mossbauer (Figure 1(1S) and Table 3) data.

Rietveld refinement (Table 2) and XRD pattern (Figure 2) data showed that the presence of seawater 4.0 Gy made the gypsum phase appear. For the MG4CN sample refinement, which was synthesized in the presence of seawater and cyanide, the gypsum phase was not used in the Rietveld refinement data. However, in addition to the FT-IR spectrum, there was evidence of gypsum phase formation through a slight baseline deformation near 12.9° 2θ, the highest intensity peak of this phase. Comparing the MGSCN and MG4SCN patterns, where the goethite phase was present, it was observed that the magnetite/goethite ratio was higher in the MGSCN sample (Table 2). This demonstrates that the presence of sulfur induced goethite formation, but that the presence of seawater 4.0 Gy diminished this effect through ferrihydrite formation. With respect to the effects of cyanide on MGCN and MG4CN patterns, it inhibited the formation of goethite, since the MG4P sample also presented a goethite phase in a similar proportion to that observed in the MG4SCN sample. A decrease in crystallinity was also observed in all samples synthesized in the presence of additional anions, as evidenced by the broadening of diffraction peaks in Figure 2.

**Figure 4:**
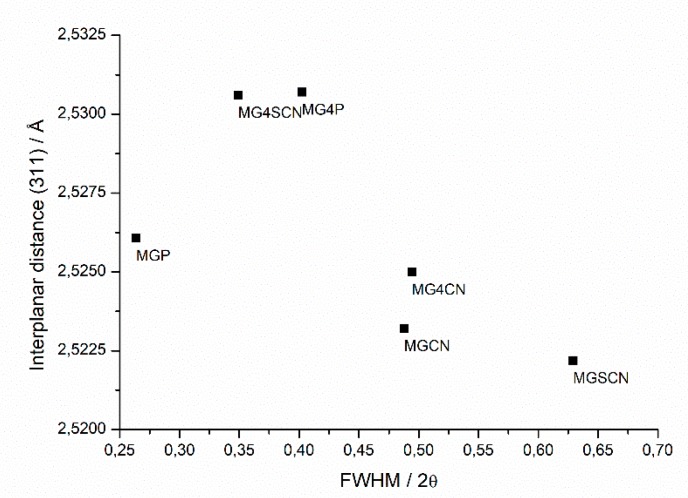
Full width at half maximum (FHWM) versus interplanar distance data of the 2θ = 41.44° peak of all magnetite samples.

Full width at half maximum (FHWM) versus interplanar distance data of the 2θ = 41.44° peak of all samples can be observed in Figure 4.

The standard magnetite sample (MGP) presented higher crystallinity and, consequently, a smaller FWHM value (Figure 4). For samples with cyanide, results indicate that this ion induced maintenance of the synthesis mechanism, supporting the magnetite phase and somehow blocking goethite formation. The direct response to the presence of cyanide in reaction medium was the decrease in mineral crystallinity (Figure 4). For the sample synthesized in the presence of a thiocyanate ion (MGSCN), a decrease in crystallinity was observed due to the increase in FWHM value. The presence of thiocyanate led to the formation of large amounts of goethite. Thus, the crystallinity of magnetite cannot only be assumed by its peak, as peak broadening may have been caused by many peaks overlapping from the goethite phase. The same occurs in the presence of seawater 4.0 Gy, but to a lesser extent.

In all samples synthesized in seawater 4.0 Gy, the gypsum phase was observed, probably due to the low solubility of calcium sulfate that eventually crystallized (Table 2). Aside from the observed minerals, a small amount of sylvite (a KCl mineral) was observed in the MG4SCN sample (Table 2).

Mössbauer spectra of the magnetite sample (MGP) at room temperature were in agreement with the data observed in several works (Figure 1(1S)) [47,48,49]. At room temperature, the magnetite spectrum showed two sextets derived from the sum of signals referring to two distinct iron sites on a magnetite structure. The first signal, with the highest hyperfine magnetic field temperature value (B_FH_ = 48.5) refers to the tetrahedral site occupied by high spin iron (III) atoms. The second signal, with the lower hyperfine magnetic field temperature (B_FH_ = 45.1), refers to the magnetic coupling of iron (II) and iron (III) centers, coordinated by the oxygen bridge. This coupling can be better understood as a mixed valence (2.5) of iron atoms at octahedral sites [39,40]. In the MGP and MGCN samples, only two sextets were observed, showing that the only magnetite mineral was formed and corroborating the FTIR-ATR and XRD analysis. For the MG4CN sample, typical Fe^3+^ sites of the 2-line ferrihydrite mineral were found, with isomeric shift values close to 0.3 mm s^−1^ and quadrupole splitting close to 0.7 mm s^−1^ [40]. Since ferrihydrite has an amorphous structure, XRD analyses are not expected to be highly effective in providing evidence on this mineral phase [50,51]. However, a slight decrease in the diffractogram scale showed two peaks close to 35 2θ and 62 2θ that were characteristic of ferrihydrite (Figure S2 [50,51,52]. The MG4P, MGSCN, and MG4SCN samples also showed typical ferrihydrite doublets and baseline curves on diffractograms in the same regions. This demonstrates that in addition to goethite, there was formation of ferrihydrite in some synthesis. The Mössbauer spectra of all other samples showed slight distortion in the second sextet of the magnetite signal. This result can be explained by the formation of a small amount of goethite mineral. It should be noted that for goethite, Mössbauer spectra are also a sextet at room temperature. Since the formation of the goethite phase can occur on the surface of the magnetite, the parameters of the second sextet (i.e., the outer octahedral layer) undergo major alterations, such as a slight decrease in B_FH_ values. [40,47,48] Mössbauer parameters of the MGSCN sample showed a signal referring to the goethite mineral phase that was presented in the spectra as a sextet with isomeric shift at 0.39 mm s^−1^ and quadrupole splitting at −0.23. mm s^−1^. This result is due to the formation of goethite crystals with small sizes, which generated differentiation in quadrupole splitting and hyperfine magnetic field parameters [40,47,48].

In summary, all the above results demonstrate that cyanide does not have great relevance in terms of different mineral phase formations. However, it may have acted as an inducing agent in the formation of magnetite. The presence of the thiocyanate ion induces the formation of goethite, as shown in the literature [40,41,42,43]. For the samples synthesized in seawater 4.0 Gy, besides the magnetite mineral, goethite, and/or ferrihydrite were found. This result is important for prebiotic chemistry since it shows that even under anoxic conditions and without UV-radiation the formation of these minerals can be possible under prebiotic conditions

According to data in the literature, magnetite has pH_PZC_ values from 6.0 to 7.0 [39,43]. The MGP and MGCN samples were the only samples that exhibited a magnetite phase, and were the only ones with pH_PZC_ values in the ranges shown for magnetite (Table 4). The MGSCN sample had a higher pH_PCZ_ value than expected for magnetite samples. Apparently, the formation of goethite in the sample increased the pH_PZC_ value (Table 4). It should be noted that goethite has pH_PZC_ values close to 7.5 [43]. For the samples MG4P and MG4CN, the presence of goethite/ferrihydrite and ferrihydrite (Table 3), whose pH_PZC_ values range from 7.5 to 9.0, caused a natural increase in the pH_PZC_ values. These values were in agreement with the data obtained from the FTIR-ATR, Mössbauer, and XRD analyses.

All transmission electron microscopy (TEM) images showed typical octahedral crystals of magnetite in different sizes (Figure 3) [44]. It was also possible to observe small stick-shaped crystals in some samples that were typical of goethite formation. TEM images of MGP and MGCN samples showed octahedral shapes of crystalline magnetite with sizes close to 50 nm. Magnification of the images demonstrated the diffraction fringes of the different crystalline planes and their respective interplanar distance values. For all samples, the values obtained from the XRD analyses corroborated the results found in the microscopy images. The images obtained for the MG4SCN, MG4P, and MGSCN samples had, besides magnetite octahedral crystals, small goethite crystals and other needle or leaf-shaped structures. These two structures can be attributed, respectively, to the formation of goethite and ferrihydrite.

Comparing magnetite crystal images for all samples, a decrease in crystallinity was noticeable for MG4P, MGSCN, and MG4SCN samples, especially when observing the edges of the crystals of the images (see the MG4P, MGSCN, and MG4SCN samples in Figure 3). The results of the TEM showed that there was a direct correlation with the data obtained from XRD diffraction. In short, images obtained by transmission electron microscopy showed that there was indeed a correlation among X-ray diffraction, Mössbauer, and FTIR-ATR analyses with the formation of different minerals and different crystallinity. It is noticeable from the TEM images that the MGP and MGCN samples, whose unique composition is magnetite, presented better crystallinity when compared to other samples. When the other samples are highlighted, it is possible to observe different mineral phases formed together with magnetite. Thus, it can be confirmed that thiocyanate ions and those present in seawater 4.0 Gy had an effect on the formation of other mineral phases and that cyanide ions can only influence the crystallinity of previously formed magnetite or inhibit the formation of goethite.

Finally, cyanide and thiocyanate quantification results showed no significant consumption of these anions in the magnetite synthesis. In addition, cyanide and thiocyanate had little adsorption (not significant in spectroscopy measurements) onto magnetite, even after the pH of the solution changed to below pH_PZC_ values of magnetite. This indicates that their adsorption was thermodynamically unfavorable to these minerals. However, according to all previously shown results they play a very important role in iron oxide synthesis mechanisms, mainly through goethite and ferrihydrite formation without oxygen or UV radiation. In accordance with Millero et al., in solutions with a pH higher than 3.5, iron(II) is oxidized to metastable iron(III) hydroxide as ferrihydrite, which then dehydrates to goethite or hematite [44]. Thus, iron(II) coordination with cyanide/thiocyanate/ion seawater changes the redox potential of this reaction and directs the reaction to the formation of different products. Summarizing this upshot, cyanide coordination with iron(II) leads to magnetite upkeep, thiocyanate leads to goethite formation, and seawater 4.0 Gy ions, such as sulfate, lead to goethite and ferrihydrite production.

### Implications for Prebiotic Chemistry

Magnetite must have played an important role in the emergence of life on Earth. It has been shown that the synthesis of this mineral can lead to the formation of important molecules in prebiotic terms, as well as acting as a catalyst in Fischer—Tropsch synthesis (FTS). Since primitive Earth had a large amount of Fe^2+^ solubilized in its primitive oceans, several molecules and dissolved ions, such as cyanide, thiocyanate, sulfate and nitrate, must have interacted with these ferrous ions and led to the formation of different minerals or molecules, agreeing with the Bernal hypothesis [45]. Nitrate played an important role in magnetite synthesis. The concentration of nitrate used in the experiments is much higher than probably in the prebiotic seas (< 1 μmol L^−1^) [53]. Ranjan et al. also suggested that nitrate concentration could be higher than 1 μmol L^−1^ in prebiotic pounds [53] Previous studies showed that depending on the concentration of sulfide, the concentration of iron in the early oceans could reach a value around 100 μmol L^−1^ [54,55,56]. As the concentration of iron used in our experiments is proportionally higher than nitrate, it is assumed that the experiments have plausibility in terms of prebiotic chemistry experiments. Variations in the methodology of magnetite synthesis, mainly through the addition of cyanide and thiocyanate to the reaction medium, with and without seawater 4.0 Gy, had an effect on the product formed from the Fe^2+^ oxidation reaction. Furthermore, magnetite is considered an important prebiotic mineral and other minerals are formed from the insertion of new reaction parameters. Cyanide, one of the most important building blocks of prebiotic chemistry, has been shown to be an effective protective agent in magnetite formation, probably from Fe^2+^ ion stabilization. In the meantime, aside from magnetite, the addition of seawater 4.0 Gy to MG4P, MG4CN, and MG4SCN samples was shown to produce ferrihydrite. Only the MG4SCN sample presented a small amount of gypsum formed. The insertion of thiocyanate in the reaction seemed to promote the formation of goethite, a fact that was already expected since the synthesis of iron minerals performed in the presence of sulfur compounds tends to form this mineral.

The fact that different Fe^3+^ containing minerals are formed from Fe^2+^ solutions indicates that, in prebiotic environments, minerals containing only Fe^3+^ centers such as ferrihydrite and goethite may also have occurred. Unlike the premise that Fe^3+^ minerals were formed only after the great oxygenation of the planet due to the appearance of early life forms, the concept that different molecules have a direct influence on the formation of ferric minerals cannot be ruled out. It is probable that small molecules such as cyanide and thiocyanate played an important role in the formation of other mineral phases from the precipitation of Fe^2+^ hydroxide. In addition, the results show that the composition of water in the early oceans should not be neglected in prebiotic chemistry experiments, since other mineral phases can be formed when the synthesis is performed solely by the addition of salts that made up the oceans 4 billion years ago.

## 5. Conclusions

Through the synthesis of magnetite samples under different conditions in the presence of cyanide and thiocyanate and the data analysis of magnetite samples according to different characterization techniques, one can infer three main assertions: (i) The presence of cyanide leads to the protection of the formation of magnetite from Fe^2+^ solutions. TEM images and XRD patterns showed high crystallinity of magnetite samples and the absence of goethite. (ii) The presence of thiocyanate, in contrast, leads to the formation of large amounts of goethite even under conditions without oxygen. (iii) Experiments carried out in seawater 4.0 Gy, whose major composition is calcium/magnesium/sulfate, show that these ions play an important role in the formation of goethite and ferrihydrite phases.

## Figures and Tables

**Table 1:** Reagents used in the SOLUTION 1 composition for the different synthesized samples.

Sample Code	SOLUTION 1 Reagents
MGP	5.72 g (30 mmol) of FeCl_2_·7H_2_O/60 mL ultrapure water
MG4P	5.72 g (30 mmol) of FeCl_2_·7H_2_O/60 mL of seawater 4.0 Gy
MGCN	5.72 g (30 mmol) of FeCl_2_·7H_2_O)/60 mL ultrapure water/3.9 g (60 mmol) of KCN
MG4CN	5.72 g (30 mmol) of FeCl_2_·7H_2_O/60 mL of seawater 4.0 Gy/3.9 g (60 mmol) of KCN
MGSCN	5.72 g (30 mmol) of FeCl_2_·7H_2_O)/60 mL ultra-pure water/5.82 g (60 mmol) of KSCN
MG4SCN	5.72 g (30 mmol) of FeCl_2_·7H_2_O/60 mL of seawater 4.0 Gy/5.82 g (60 mmol) of KSCN

60 mL of seawater 4.0 Gy composition (mg): Na_2_SO_4_ (16.2); MgCl_2_·6H_2_O (30.0); CaCl_2_·2H_2_O (150.0); KBr (3.0); K_2_SO_4_ (24.0); MgSO_4_ (900.0) [30]. KCN-potassium cyanide, KSCN-potassium thiocyanateEach synthesis was performed at least six times.

**Table 2:** Selected Rietveld parameters from XRD patterns of all solid samples and mineral phases found in the respective samples.

Sample	Mineral Phase Found	% = (Mineral Phase Mass/Total Mass) × 100	R_wp_/%	χ^2^
MGP	Magnetite	100	22.16196	2.59452
MG4P	Magnetite	53.7	22.28601	2.46596
	Goethite	26.9		
	Gypsum	19.4		
MGCN	Magnetite	100	20.11383	1.70119
MG4CN	Magnetite	100	24.34639	2.90163
MGSCN	Magnetite	59.4	23.78344	3.03894
	Goethite	40.6		
MG4SCN	Magnetite	49.5	19.47453	2.09246
	Goethite	36.2		
	Gypsum	10.1		
	Sylvite	4.3		

**Table 3:** Mössbauerspectra parameters of all solid samples and iron mineral correspondence. (IS: Isomeric shift; QS: Quadrupole splitting; B_HF_: Magnetic hyperfine field).

Sample	Sub-Spectra	IS/mm s^−1^	QS/mm s^−1^	B_hf_/T	Mineral Correspondence
MGP	Sextet	0.34	−0.08	48.5	Magnetite
	Sextet	0.65	−0.01	45.1	
MG4P	Sextet	0.31	−0.00	47.8	Magnetite
	* Dist.	0.63	−0.12	42.8	Magnetite/Goethite
	Sextet				
	Doublet	0.38	0.77	-----	Ferrihydrite
MGCN	Sextet	0.34	−0.08	51.2	Magnetite
	Sextet	0.67	−0.10	47.9	
MG4CN	Sextet	0.29	−0.02	49.1	Magnetite
	Sextet	0.58	0.08	44.8	Magnetite
	Doublet	0.36	0.71	-----	Ferrihydrite
MGSCN	Sextet	0.34	−0.04	51.2	Magnetite
	Sextet	0.67	−0.06	47.4	
	* Dist.	0.34	−0.02	33.6	Goethite
	Sextet				
	Doublet	0.37	0.70	-----	Ferrihydrite
	Sextet	0.39	−0.23	39.6	Goethite
MG4SCN	Sexto	0.28	−0.03	49.2	Magnetite
	* Dist.	0.51	0.10	44.1	Magnetite/Goethite
	Sextet				
	Doublet	0.36	0.69	-----	Ferrihydrite

* Distortion of the sextet, MGP and MGCN presented the lowest values of pH_PZC_, close to neutral pH, while other samples presented values higher than 8 (Table 4).

**Table 4:** Experimental pH_PZC_ values of all solid samples.

Sample	pH_PZC_
MGP	7.34 ± 0.04
MG4P	8.97 ± 0.07
MGCN	6.15 ± 0.08
MG4CN	9.14 ± 0.04
MGSCN	8.35 ± 0.06
MG4SCN	8.79 ± 0.09

Results are shown as mean ± standard derivation. Each value represents the mean of experiments in triplicate.

## Supplementary Materials

The following are available online at https://www.mdpi.com/2075-1729/10/4/34/s1, Figure S1: Mössbauer spectra of all 26 samples. Figure S2: Amplification of XRD patterns from MG4P, MGSCN and MG4SCN samples and corresponding baseline curves (dashed line) from ferrihydrite formation. Table S1: Comments on all experiments and respective reagents. Table S2: FTIR-ATR results of some experiments. Table S3: Composition of artificial seawater 4.0 Gy.
