# Supplementary material for: Magnetite Synthesis in the Presence of Cyanide or Thiocyanate under Prebiotic Chemistry Conditions
Source: Life (Basel). 2020 Apr 2;10(4):34. doi: 10.3390/life10040034 (PMC7236013; doi:10.3390/life10040034)
Supplement: Supplementary file 1 [file life-10-00034-s001.pdf]

life

Supplementary Material

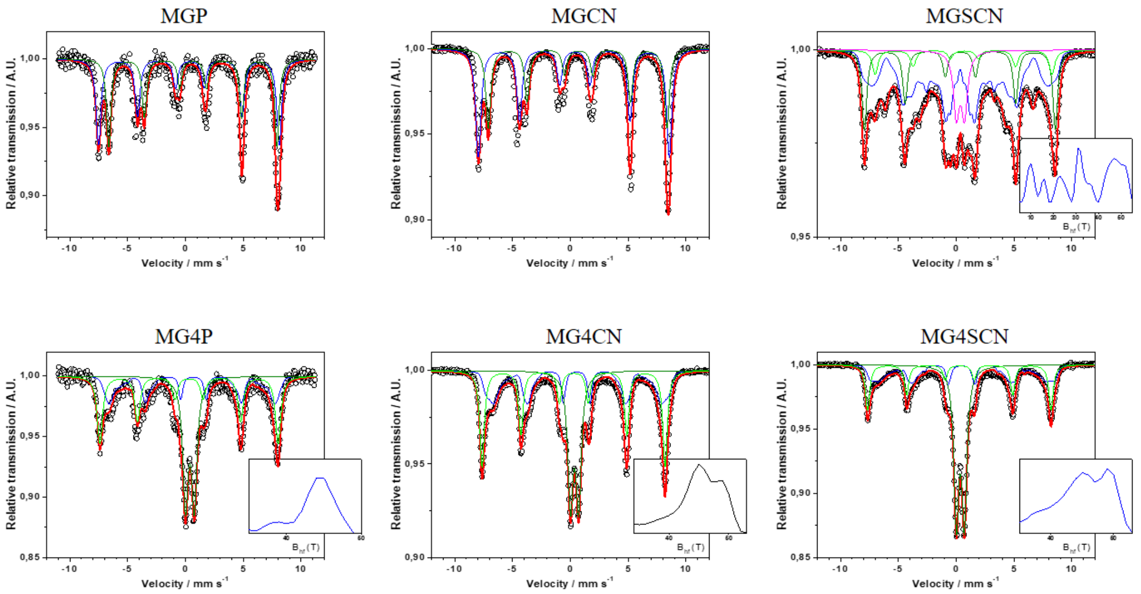

Figure S1. Mössbauer spectra of all 26 samples.

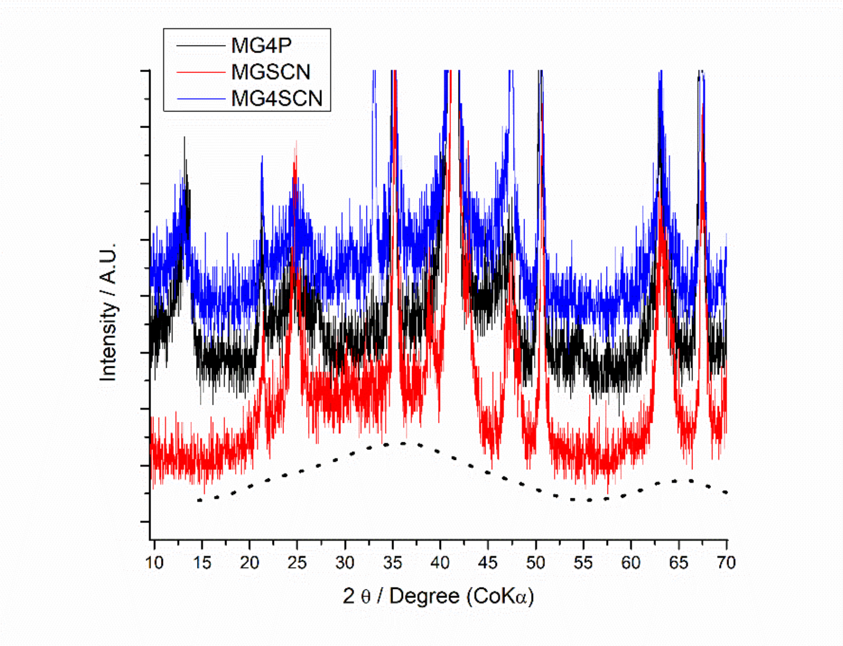

Figure S2. Amplification of XRD patterns from MG4P, MGSCN and MG4SCN samples and corresponding baseline curves (dashed line) from ferrihydrite formation.

Control Experiments

In order to show that the syntheses were carried out under anoxic conditions, control experiments were performed. Since Fe^{2+} is easily oxidized to Fe^{3+}, experiments were performed to ensure that contaminants or oxygen did not interfere with the observed products. For these experiments, the same concentrations and conditions were used as mentioned in the text. These were performed in ultrapure water and seawater 4.0 Gy, to show that magnetite, under these conditions, is formed only in the simultaneous presence of KOH and KNO_3. Thus, we carried out the experiments

with the separate addition of these two reagents. Table 1S shows the conditions under which the experiments were carried out. The experiments were monitored by FTIR spectroscopy to be sure of what was produced in each situation (Table S2).

Table S1. Comments on all experiments and respective reagents.

Experiment Number	Reagents	Comments
1	Fe^{2+} solution in ultrapure water	No color change or precipitate formation
2	Fe^{2+} solution in seawater 4.0 Gy	No color change or precipitate formation
3	Fe^{2+} solution in ultrapure water + KNO_3	No color change or precipitate formation
4	Fe^{2+} solution in seawater 4.0 Gy + KNO_3	No color change or precipitate formation
5	Fe^{2+} solution in ultrapure water + KOH	Dark blue-green precipitate
6	Fe^{2+} solution in seawater 4.0 Gy + KOH	Dark blue-green precipitate
7	Fe^{2+} solution in ultrapure water + KCN	Dark yellow precipitate
8	Fe^{2+} solution in seawater 4.0 Gy + KCN	Dark yellow precipitate
9	Fe^{2+} solution in ultrapure water + KCN + KNO_3	Dark yellow precipitate
10	Fe^{2+} solution in seawater 4.0 Gy + KCN + KNO_3	Dark yellow precipitate
11	Fe^{2+} solution in ultrapure water + KCN + KOH	Dark blue-green precipitate
12	Fe^{2+} solution in seawater 4.0 Gy + KCN + KOH	Dark blue-green precipitate
13	Fe^{2+} solution in ultrapure water + KSCN	Color turns to light red and no precipitate formation
14	Fe^{2+} solution in seawater 4.0 Gy + KSCN	Color turns to light red and no precipitate formation
15	Fe^{2+} solution in ultrapure water + KSCN + KNO_3	Color turns to light red and no precipitate formation
16	Fe^{2+} solution in seawater 4.0 Gy + KSCN + KNO_3	Color turns to light red and no precipitate formation
17	Fe^{2+} solution in ultrapure water + KSCN + KOH	Dark blue-green precipitate
18	Fe^{2+} solution in seawater 4.0 Gy + KSCN + KOH	Dark blue-green precipitate

Infrared spectra of the solids did not show the formation of oxidized iron oxides/hydroxides (Figure 1, Table S2). In all cases, the pH increases by KOH addition led to the formation of green rust. KNO_3 did not appear to interfere when added without increasing pH. Experiments carried out with cyanide and thiocyanate showed that different intermediates were formed and influenced the final mineral products as described in the original text.

Blue color precipitates presented a broad band at 420 cm^{-1} and a small band around 500 cm^{-1}. These stretching frequencies were linked to the green rust formation and were assigned to $Fe^{(II)}$-O stretching [1]. Dark yellow product, formed after KCN addition onto Fe^{2+} solution, showed broad bands at 2064 cm^{-1} assigned to CN stretching, with a small band at 580 cm^{-1} assigned to Fe-C(cyanide) stretching [2,3]. Experiments performed with KSCN showed a band at 2062 cm^{-1} attributed to CN stretching from SCN^- ions [2,3]. All experiments performed with seawater 4.0 Gy showed a band around 1090 cm^{-1} typical of ionic SO_4^{2-} presence [4]. In another way, experiments performed with KNO_3 showed a band around 1360 cm^{-1} typical of NO_3^{2-} presence [1]. Experiments without color change or precipitate formation were not subject to FTIR-ATR analysis.

Table S2. FTIR-ATR results of some experiments.

Experiment Number	Comments	Ftir-Atr Band (CM^{-1})	Conclusion
5	Dark blue-green precipitate	420, ~500, ~3450	Green-Rust formation ($Fe(OH)_2$)
6	Dark blue-green precipitate	420, ~500, 1090, ~3450	Green-Rust formation ($Fe(OH)_2$)
7	Dark yellow precipitate	580, 2062	Fe(II)-CN
8	Dark yellow precipitate	580, 1090, 2062	Fe(II)-CN
9	Dark yellow precipitate	580, 1360, 2062	Fe(II)-CN
10	Dark yellow precipitate	580, 1090, 1360, 2062	Fe(II)-CN
11	Dark blue-green precipitate	420, ~500, 2060, ~3450	Green-Rust formation ($Fe(OH)_2$) and Fe(II)-CN bond
12	Dark blue-green precipitate	420, ~500, 1090, 2060, ~3450	Green-Rust formation ($Fe(OH)_2$) and Fe(II)-CN bond
17	Dark blue-green precipitate	420, ~500, 2058, ~3450	Green-Rust formation ($Fe(OH)_2$) and free SCN^-
18	Dark blue-green precipitate	420, ~500, 1090, 2058, ~3450	Green-Rust formation ($Fe(OH)_2$) and free SCN^-

Results showed that the techniques used were reliable in order to prevent the oxidation of Fe^{2+} in solution. The addition of NO_3^{2-} ions did not influence the final products and did not lead to iron oxidation. The presence of cyanide led to the formation of Fe $(CN)_2$ and other Fe-CN complexes, as was proven by the FTIR analyses. After the addition of KOH, the precipitate changed color to blue, showing that, even in the presence of cyanide, there was formation of green rust. The same was observed for the addition of KSCN.

Table S3. Composition of artificial seawater 4.0 Gy.

Salt*	Mass (g)
Na_2SO_4	0.271
$MgCl_2.6H_2O$	0.500
$CaCl_2$ $2H_2O$	2.50
KBr	0.050
K_2SO_4	0.400
$MgSO_4$	15.00

*Each salt should be added in the order in which they are shown [5].

References

[1] Cornell, R. M.; Schwertmann, U. *The Iron Oxides: Structure, Properties, Reactions, Occurrences and Uses*, 2nd ed.; Wiley: Hoboken, New Jersey, 2003.

[2] Nakamoto, K. *Infrared and Raman Spectra of Inorganic and Coordination Compounds. Part B: Applications in Coordination, Organometallic, and Bioinorganic Chemistry*, 6th ed.; Wiley: Hoboken, New Jersey, 2009.

[3] Colthup, N.B., Daly, L.H., Wiberley, S.E. *Infrared and Raman Spectroscopy*, 3rd ed; Academic Press Inc.: Boston, MA, USA, 1990.

[4] Peak, D., Ford, R.G., Sparks D.L. An in situ ATR-FTIR investigation of sulfate bonding mechanisms on goethite. *J. Coll. Interf. Sci.* **1999**, *218*, 289–299. https://doi.org/10.1006/jcis.1999.6405

[5] Zaia, D. A. M. Adsorption of Amino Acids and Nucleic Acid Bases onto Minerals: A Few Suggestions for Prebiotic Chemistry Experiments. *Int. J. Astrobiol.* **2012**, *11*, 229–234. https://doi.org/10.1017/S1473550412000195.
